# Selection of Treatment Strategies among Patients with Type 2 Diabetes Mellitus in Malaysia: A Grounded Theory Approach

**DOI:** 10.1371/journal.pone.0147127

**Published:** 2016-01-26

**Authors:** Lee Lan Low, Seng Fah Tong, Wah Yun Low

**Affiliations:** 1 Medical Education and Research Development Unit, Faculty of Medicine, University of Malaya, Kuala Lumpur, Malaysia; 2 Institute for Health Systems Research, Ministry of Health Malaysia, Selangor, Malaysia; 3 Department of Family Medicine, Faculty of Medicine, Universiti Kebangsaan Malaysia, Kuala Lumpur, Malaysia; 4 Research and Management Center, Faculty of Medicine, University of Malaya, Kuala Lumpur, Malaysia; University of Tolima, COLOMBIA

## Abstract

**Background:**

Diabetes Mellitus is a multifaceted chronic illness and its life-long treatment process requires patients to continuously engage with the healthcare system. The understanding of how patients manoeuvre through the healthcare system for treatment is crucial in assisting them to optimise their disease management. This study aims to explore issues determining patients’ treatment strategies and the process of patients manoeuvring through the current healthcare system in selecting their choice of treatment for T2DM.

**Methods:**

The Grounded Theory methodology was used. Twelve patients with Type 2 Diabetes Mellitus, nine family members and five healthcare providers from the primary care clinics were interviewed using a semi-structured interview guide. Three focus group discussions were conducted among thirteen healthcare providers from public primary care clinics. Both purposive and theoretical samplings were used for data collection. The interviews were audio-taped and transcribed verbatim, followed by line-by-line coding and constant comparison to identify the categories and core category.

**Results:**

The concept of “experimentation” was observed in patients’ help-seeking behaviour. The “experimentation” process required triggers, followed by information seeking related to treatment characteristics from trusted family members, friends and healthcare providers to enable decisions to be made on the choice of treatment modalities. The whole process was dynamic and iterative through interaction with the healthcare system. The decision-making process in choosing the types of treatment was complex with an element of trial-and-error. The anchor of this process was the desire to fulfil the patient’s expected outcome.

**Conclusion:**

Patients with Type 2 Diabetes Mellitus continuously used “experimentation” in their treatment strategies and help-seeking process. The “experimentation” process was experiential, with continuous evaluation, information seeking and decision-making tinged with the element of trial-and-error. The theoretical model generated from this study is abstract, is believed to have a broad applicability to other diseases, may be applied at varying stages of disease development and is non-context specific.

## Introduction

Type 2 Diabetes Mellitus (T2DM), a chronic disease affecting millions of adults globally, becoming a major burden to the healthcare system in many countries [1]. Diabetes Mellitus is also a major concern in Malaysia where the prevalence of T2DM among adults aged 30 years and above has increased from 14.9% in 2006 to 20.8% in 2011[2,3]. The lifelong process of treatment for T2DM can be expensive and burdensome to patients and the country. The economic burden of diabetes treatment is substantial and the estimated direct cost for outpatient treatment is USD77.71per patient per annum [4]. Treating a chronic disease such as diabetes costs 66% more than the treatment for a fever [5].

Like many other chronic diseases, T2DM is a difficult illness to live with because of the complexity of treatment, the requirement of strong patient commitment and continuous adherence to the treatment plan. Studies have shown that effective diabetes care is multidimensional, involving a multispecialty healthcare team and a patient’s self-care approach [6–8]. Diabetes care can have a great impact on the patient’s quality of life, as it requires a change in lifestyle and compliance to the treatment plan. Patient with T2DM, often strive to improve their quality of life and reduce diabetes related complications [9,10]. Their efforts to strive for a balance in daily life without any symptoms are reflected in their help-seeking behaviour; i.e. their utilization of health services [11]. Failure to achieve an optimal balance in the quality of their daily life often results in poor help seeking which leads to poor diabetic control. Hence, an understanding of patient’s help-seeking behaviour may offer a solution for better diabetic control.

Although help-seeking behaviour studies related to diabetes have been conducted in many settings, most of these studies were exploring the types of treatments [12,13] that patients engaged in, or the places where they sought care [11,14], and the barriers and facilitators of access to diabetes treatment [15,16]. However, the patient’s help-seeking pattern extends beyond these variables. Help-seeking is a complex process which involves interplay of the many linked variables, such as predisposing factors, the condition of the illness, the healthcare system and others [17,18]. Each variable that determines help-seeking has a different influencing factor on the help-seeking process. Hence, an understanding of how patients with T2DM manoeuvre through the healthcare system for their diabetes treatment can assist the healthcare system in a country in reacting and facilitating the process of disease management. The healthcare system in this study refers to all activities involved in the delivery of promoting, restoring and maintaining the health of the population, including those provided by modern (public and private healthcare services) and traditional and complementary medicine [19].

To our knowledge, there is no model or study that has been carried out so far in Malaysia that describes the help-seeking process among patients with T2DM. The aim of this study is to explore issues determining patients’ treatment strategies and the process of patients manoeuvre through the current healthcare system in selecting their choice of treatment for T2DM. Through the understanding of the process of selection of treatment strategies among patients with T2DM and underlying issues, shortfalls can be identified to support the development of more comprehensive and holistic intervention programs for patients with T2DM.

## Methods

In line with the objective of exploring the process of patients with T2DM manoeuvre through the Malaysian healthcare system, the grounded theory methodology was adopted [20], with an anticipation of developing a substantive theoretical model to explain the help-seeking behaviour or treatment strategies among patients with T2DM. The grounded theory approach has an added advantage that it allows analysis beyond theme generation. It offers a technique to examine the relationship between the variables depicting the dynamic process of help-seeking.

In this study, two data collection techniques were used; in-depth interviews (IDIs) among patients with T2DM, family members and the healthcare providers and focus ground discussions (FGDs) among the healthcare providers from the public primary care settings.

### Sampling and recruitment of participants

The study was carried out in primary care settings: two public primary healthcare centres and five private general practitioners clinics in the state of Selangor, Malaysia. Purposive sampling strategy was employed initially to recruit patients for the study with the intention of capturing a range of patients with varying social backgrounds, both from the public and private primary care settings. Subsequently, theoretical sampling was applied to aid saturation of the theoretical framework in explaining the help-seeking behaviour of the patients. Theoretical sampling was used simultaneously at the time of data collection, coding and analysis. The family members and healthcare providers were included in exploring the influence of people surrounding patients and their role in the process of developing a theory[21]. For example, about midway through the study it became evident from the preliminary analysis that participants (patients) were sharing their experiences, how they were influenced by family members, friends and healthcare providers in their daily diabetes management, particularly in information seeking on diabetes care. This subsequently led to the identification of the “core category” for this study and achieved theoretical saturation.

The recruitment process started by approaching Malaysians aged 30 years and above who were diagnosed with T2DM for more than two years and who had sought treatment for diabetes at primary care clinics. A two-year cut off point was used to ensure that they were engaged in certain behaviours of help-seeking, especially for chronic illnesses where self-management is important. Patients from the private primary care clinics were introduced by the general practitioners. Upon getting their consent, the date, time and the venues for the interviews were decided based on the patients’ convenience. Briefing about the study was given personally to those from public facilities and through phone calls for those from private primary care clinics. Family members, on the other hand, were introduced by participants (patients) and contact numbers were obtained for future communication regarding the objective and arrangement of the interview sessions. However, three male participants refused to give their family members’ contact numbers for the interviews, citing inconveniences as the reason.

For the three FGDs, the recruitment of participants included various categories and speciality disciplines of healthcare providers from public primary care clinics. Assistance from the clinic nurses was obtained to help recruit and arrange the FGD sessions. The nurses were briefed about the project and the organization of the FGDs.

The private primary care doctors were interviewed face-to-face at their clinics. Although, the interview sessions were challenging due to time constraints, all general practitioners selected for this study were cooperative.

### Data collection

Data was obtained through IDIs and FGDs. Twenty six IDIs, comprising twelve patients with T2DM, nine family members of the patients and five primary care doctors were conducted. Three FGD sessions were held among thirteen healthcare providers from the public primary care setting. FGDs could not be carried out for the private healthcare providers due to difficulty in finding a suitable time to convene a discussion.

Data was collected from September 2012 to May 2013 by the first author (LLL). Interview guides were developed along with a list of questions and prompts to clarify and elicit more detailed information. The guides covered the participants’ experiences on how their T2DM was diagnosed and treated, their perceptions about T2DM, the treatment they undertook, what made them choose the desired treatment and, how T2DM affected their daily lives. Family members were asked about their experiences living with patients with T2DM, their own perceptions, and their role in supporting the management of diabetes among their family members. The healthcare providers were interviewed about their experiences and perceptions of treating patients with T2DM and patients’ attitude and behaviour towards treatment and management of diabetes. In order to stimulate a broad and in-depth discussion, different categories and speciality disciplines of healthcare providers comprising medical officers, a dietician, a pharmacist, medical assistants, nurses and assistant pharmacists from the public primary care setting were included in the focus group discussions. The duration of IDIs ranged from 30 to 60 minutes while FGDs lasted from one to one and a half hours. Participant information sheets were distributed prior to the interview sessions and written informed consent was obtained from all participants, with assurance given of confidentiality and that data would be used only for the study. Languages of interviews were based on the participant’s level of understanding of the English, Malay or Chinese languages. The IDIs and FGDs were audio-taped and transcribed verbatim.

In appreciation, all participants were given a token of RM30 (Ringgit Malaysia) after completion of the interviews or FGD sessions. All identifiers to the participants were given a code to maintain the anonymous of the transcripts prior to interpretation.

### Data management and analysis

Data management was facilitated by Nvivo 10 (Qualitative data analysis computer software). The analyses were completed primarily by the first author, followed by frequent discussions with the second and third authors to receive feedback on the emerging themes. The interviews conducted in the Malay language were transcribed without translation; however the interviews in the Chinese language were transcribed and translated to English for analysis. The analysis was done bilingually. The text was read in its original language and coded in English. The quotes in Malay language were translated into English for the purpose of publication.

Data analysis began after the first interview. The data were analysed according to the grounded theory methods [21]. The texts were read a few times before coding began. Sections of the text were coded, line-by-line as these were considered significant for the first three interview transcripts with patients. Codes with similar concepts were reviewed and clustered into categories and subcategories. The categories and sub-categories were identified according to the similarities and differences in line-by-line codes. While coding, constant comparison was made within and between categories, and between categories and texts subsumed under the same categories. In between line-by-line coding, memos were also written to capture the moments of thoughts. The idea, curiosity, and the flow emerging during the analytical interpretation of data were captured in memos by a sketching and sorting process, especially in the attempt to capture the help-seeking process. Identification of categories and subcategories was facilitated by discussion with co-authors and further defined. The subsequent verbatim texts were analysed using the initial categories as guides. Constant reflection, modification and adjustment to the categories and subcategories were carried out to identify the best fit categories. Saturation of categories was achieved through the theoretical sampling as described above.

The core category “experimentation” was identified based on its ability to subsume main categories explaining the underpinnings of patients’ help-seeking behaviour. Saturation was observed and achieved after twenty-nine transcripts consisting of 26 IDIs and 3 FGDs where no new categories were found to add further to the understanding of the patients’ help-seeking process. A substantive model was then developed and presented to participants in the study to gain feedback and to test the validity of data analysis.

### Ethical Consideration

This study was approved by the Institutional Review Board at the Institute for Health Systems Research, Ministry of Health Malaysia and the Medical Research and Ethical Committee (MREC), Ministry of Health Malaysia (NMRR-12-457-12193). Participants were provided with full explanation and a written information sheet prior to the IDIs and FGDs. Informed consent and permission to audio record the interview sessions were obtained from all participants.

## Results

The data were generated from in-depth interviews and focus group discussions among patients with T2DM, their family members and healthcare providers from two public primary care clinics and five general practitioners. The socio-demographic characteristics of patients with T2DM, family members and healthcare providers are shown in Tables 1 and 2.

**Table 1:** Socio-demography of patients with T2DM.

Characteristics	Participants (n = 12)
Mean age (range) (years)	55 (50–62)
Sex	
Male	5
Female	7
Ethnic group	
Malay	4
Chinese	4
Indian	4
Marital Status	
Single	1
Married	9
Widow	2
Education Level	
No formal education	1
Primary school	1
Secondary school	7
Tertiary (college/university)	3
Employment	
Public employee	2
Private employee	4
Unemployed	1
Retired	1
Housewife	4
Mean years (range) diagnosed with T2DM	8.7 (2.5–21 years)

**Table 2:** Socio-demography of family members and healthcare providers (HCPs).

Characteristics	Family Members (n = 9)	HCPs (n = 18)
Age (range) (years)	24–67	23–65
Sex		
Male	2	5
Female	7	13
Education Level		
No formal education	1	-
Primary school	2	-
Secondary school	5	-
Tertiary (college/university)	1	18
Occupation (HCP)		
Doctor	-	8
Pharmacist	-	1
Nutritionist	-	1
Nurse	-	5
Medical Assistant	-	1
Pharmacist Assistant	-	2
Relationship with patients	Spouses, daughter, sister & cousin	Healthcare provider
Experience managing patients with T2DM (range) (months/years)	-	2 months to 36 yrs

### Experimenting on diabetes care—the core category

The term ‘experimenting’ was an *in vivo* code, the word used by participants to describe their treatment strategies and the help-seeking process.

“*Yes*, *I am testing*! *Actually*, *I am* ***experimenting***, *experiment*. *Yes*, *to tell you the truth*, *I am not a clever person*, *but I am experimenting with my own knowledge*.*” (Male*, *58 years old*, *diagnosed with T2DM 7 years ago)*.

**Fig 1:**
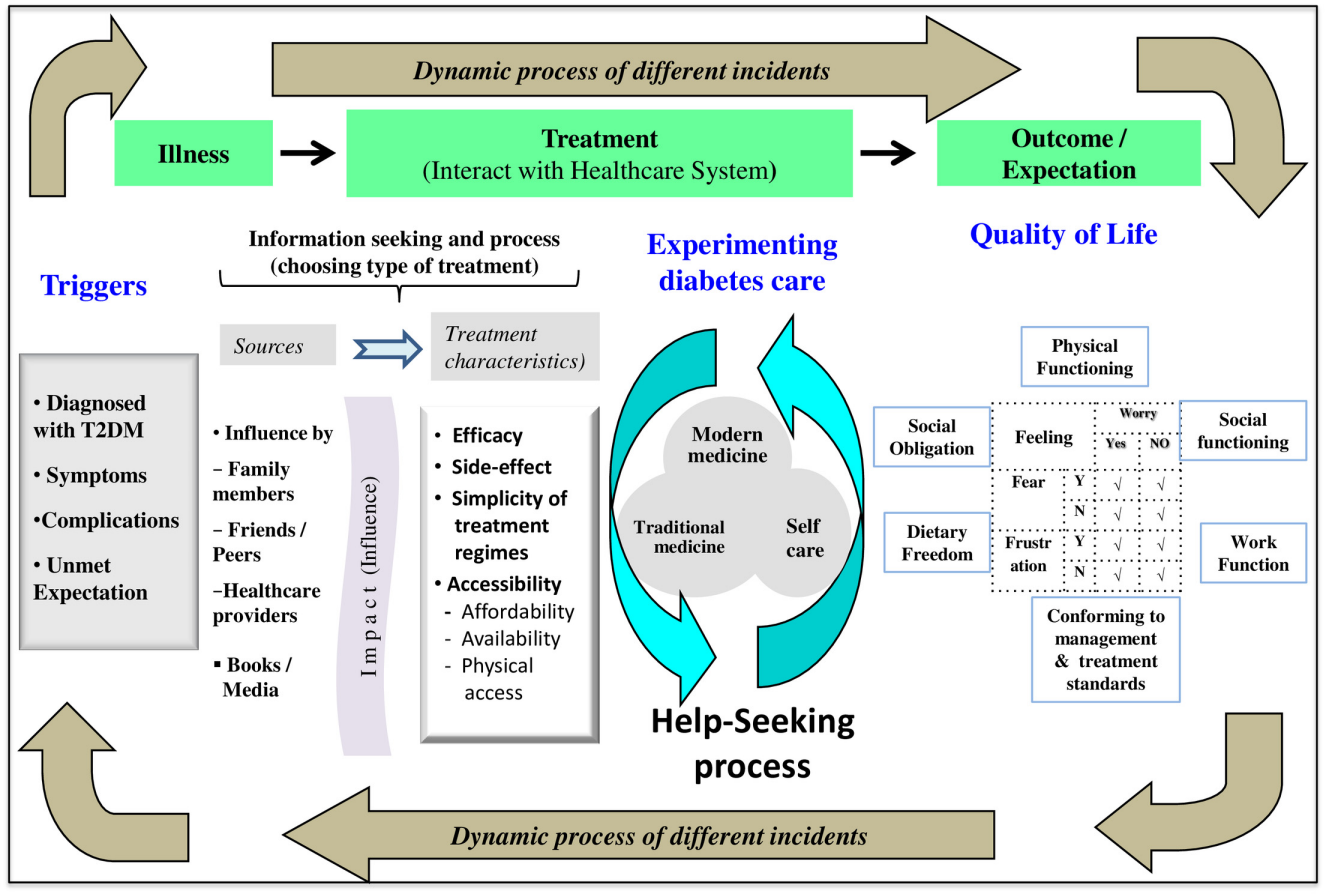
Model for help-seeking behaviour among patients with Type 2 Diabetes Mellitus in the primary care setting.

In managing diabetes, patients took an experimental approach. Patients who used the healthcare system took a loop of “trial-and-error” approach. The approach started with a trigger to try a particular type of treatment in order to eliminate the symptom(s) or fulfil an expected outcome, followed by patients’ own assessment of treatment outcomes; and finally closing the loop by a decision to keep their current treatment modality or change to another treatment modality. The “experimentation” involved trying or testing different treatment modalities besides the routine medication or treatment prescribed by the doctor. The whole process of “experimentation” is shown in Fig 1. The circle of experimentation will continue if patients were dissatisfied with their anticipated health outcomes (unmet expectation). The main reason that triggered the “experimentation” was the need to fulfil their anticipated health outcomes such as to achieve better quality of life, to overcome the signs and symptoms, avoiding later complication or deterioration—the “triggers”. Any form of “trigger” can lead to “experimentation” even if it was only to adjust the diet.

This study found that the following four determinants inter-played for the “experimentation” process to support choice of diabetes management or treatment modalities by patients with T2DM: 1) triggers, 2) the additional information to base a decision on which treatment modality to try, 3) the “experimentation” itself, and 4) the expected health outcomes of the experiment.

### Triggers

In this study “triggers” emerged with the diagnosis of diabetes and when patients had unmet expectation or when the expected outcome was not achieved. Since the study was conducted in healthcare settings, the diagnosis was usually made by healthcare providers either when patients presented with the symptoms of diabetes or through health screening. In the latter instance, it was usually asymptomatic. When the diagnosis was made, the patients would attempt to restore or maintain their quality of life by eliminating the symptoms they suffered or by improving diabetes control to avoid future complications. The excerpt below shared by one of the family members relates how worried they were upon hearing the diagnosis, which triggered the trial-and-error process.

*“*… *for me it is something unpleasant [being diagnosed with T2DM]*, *like a disaster*. *I kept asking others who had experience with diabetes*. *At the beginning I was scared*. *Everything was unexpected*. *She then tried to seek traditional medicine*. *Indeed*, *we were trying to seek traditional medicine all over the place*.*” (Patient’s husband*, *60 years old)*.

Patients may also experience the symptoms on and off, as this may be due to disease progression or uncontrolled diet intake and unhealthy lifestyle. When symptoms such as tiredness, frequent urination and thirst occur, patients began to experiment with various treatment modalities, including seeking traditional therapy besides taking diabetes medication as prescribed by their doctors.

*“When my body feels a bit uncomfortable [sign of blood sugar increase] then I ask for some [traditional remedies] from my friends who are also diabetic patients*.*” (Male*, *52 years old*, *diagnosed with T2DM 4 years ago)*.

They tried to eliminate symptoms in order to restore their quality of life as feeling sick was a major concern for all patients. Thus, in this study, the perceived symptoms or complications of diabetes were the “triggers” for the “experimentation” process.

Below is another quotation from an asymptomatic patient attempting to avoid complication or preventing the illness from deteriorating, who sought further information on complementary treatment which later lead to the “experimentation”.

*“I am worried*, *as the progression of diabetes differs every day*. *Our glucose level will shoot up if we accidentally eat something sweet*. *So I asked around*. *I heard Dr*. *X is good at making our blood circulation [detoxification] better*. *His medicine costs RM150*. *After that [taking his medicine]*, *I felt more energetic*!*” (Female*, *61 years old*, *diagnosed with T2DM 10 years ago)*.

### Searching for information and knowledge

In the “experimentation” process, patients got themselves prepared for testing and ventured into trial-and-error of different modalities of treatment, after evaluating the best possible options. Actively seeking information from various sources and exploring new treatment modalities were a part of their experimentation journey. The information often sought was treatment characteristics, such as treatment efficacy, side-effects and simplicity of treatment regimens; and accessibility comprising availability of desired treatment, cost and physical access to such care. The source of information was from social networks such as family members, friends, peers and healthcare providers. The study found that patients with T2DM actively interacted with their social networks in the process of making a decision on treatment. However, the influential impact from the social networks was dependent on the strength of their relationship, including the level of trust, and support they received. Patients also gained knowledge about diabetes through the Internet as well as by reading books and pamphlets on information related to diabetes and its treatment.

In general, there were three types of treatment modalities that patients got themselves engaged in, to achieve their desired outcomes. These were: 1) modern medicine, 2) traditional and complementary medicine, and 3) self-care. These treatment modalities were either practised concurrently, consecutively, or as a mixture of any two combinations at different time intervals such as taking both the modern and traditional treatments with a gap of one to two hours in between. Such an example was shared by one participant about his experience in using both modern and traditional medicine:

*“Usually I take it*. *I say*, *not together*. *I take the diabetes medication first*, *then*, *maybe*, *one hour later*, *I take that one [traditional herbs]*.*” (Male*, *53 years old*, *diagnosed with T2DM 13 years ago)*.

*Modern medicine* is defined in this study as biomedicine [22]. The practices of modern medicine in public and private primary care are managed by qualified medical personnel, including a pharmacist. *Traditional and complementary medicine* falls outside the spectrum of modern medicine and draws on traditional therapies, which encompass consuming traditional herbal concoctions, spiritual healing and visits to shamans (faith healers). Complementary medicine includes therapeutic modalities such as chelation and ozone therapy. *Self-care* is a term widely used in diabetes management. Many publications use the term self-care, self-management and self-monitoring interchangeably [23]. However, in this paper, self-care refers to the patients’ management of their T2DM and the illness related activities, such as monitoring of blood sugar level; adherence to medication, dietary control, and physical activities or exercising; weight management, foot care, monitoring and recognising the signs and symptoms of diabetes.

### “Experimentation” in diabetes care (help-seeking process)

The experimentation involved in the decision-making process was dynamic and iterative. The “experimentations” were experiential; they produced change(s) in a person’s help-seeking behaviour and generated a powerful effect on their daily lives. For example, a participant admitted how he had to resort to trial-and-error and claimed to have achieved some form of “success” in his own “experimentation”, for which he was satisfied since it gave him better quality of life.

*“If I take diabetic medicine*, *I cannot achieve erection*. *Actually*, *I did some experiments*, *to be truthful*. *Not the scientific method*, *but experimenting based on my knowledge*… *if I monitor my diet intake*, *then my blood sugar level reduces*… *I think I have succeeded*, *yes*, *I have succeeded [with the self experiment]*.*” (Male*, *58 years old*, *diagnosed with T2DM 7 years ago)*.

“Experimentation” was supported by evaluation and decision making processes. Patients with T2DM were exposed to various types of diabetes management, through direct and indirect influences of people around them. However, the ultimate decision on the treatment modalities was based on their personal experience, perceptions and balance or trade-offs between treatment efficacy, the side-effects, simplicity of treatment regimes, affordability, availability and physical accessibility to type of treatment. The data shows the decision-making process with the elements of trade-offs between the ideal and the practical treatment.

*“If I can go to the private clinic [it] would be easy*, *faster and communication is quite clear [able to speak with HCP in the same language/dialect]*, *but if you go to a government clinic*, *too many patients*, *they [doctors] do not explain in detail*, *but in private [clinic] we have to pay the fee*.*” (Female*, *55 years old*, *diagnosed with T2DM 8 years ago)*.

While searching for the best option for the treatment, patients were aiming for a successful treatment outcome. Treatment efficacy may be a patient’s priority in deciding treatment choice. However, issues related to affordability may prevent them from opting for certain treatments. Inability to try or continue with certain treatment modalities may take place because of financial constraints, although the treatment may have been perceived effective. The quotation below illustrates how one patient discontinued taking traditional herbs because of cost, although she perceived it as beneficial.

*“I had tried Chinese herbs*. *I can sleep very well*, *but quite expensive*. *One box [of herbs] was more than RM60*. *I took a few boxes*, *but I have to stop*, *because it was expensive for the long term [taking it]*.*” (Female*, *55 years old*, *diagnosed with T2DM 8 years ago)*.

Nonetheless, individuals prioritise health differently. Participants weighed the benefits between their health and ability to pay for treatment modalities. There were participants who considered health was important, so long as they could afford to pay and would go for the treatment they perceived as effective. This was expressed by one participant.

*“Traditional medicine is expensive*, *you know*. *People are selling [it at] like RM1000*, *RM800 or RM1500*. *The money is not an issue*, *the money will come*, *no problem*, *right*? *But I think health is more important*.*” (Male*, *53 years old*, *diagnosed with T2DM 13 years ago)*.

The side-effects from the treatment were one of the treatment modality characteristics that patients were worried about. Patients would evaluate between the treatment efficacy and its side-effects. If they perceived or had experienced negative effects from a treatment modality, they would opt for the one with less side-effect, even though the other treatment gave them a positive outcome. Below is an example of one such worry which led the patient to try other treatment modalities or reduce the frequency of intake.

*“My friends drank this kratum leave [Mitragyna speciosa [24]] everyday, but I was scared. I only took it when my body did not feel good [diabetes symptoms] then I would ask [for] some from my friends. This [kratum leaves] is good, effective, can reduce your blood sugar level, but I am scared of other side-effects [if taking it too often].” (Male, 52 years old, diagnosed with T2DM 4 years ago)*.

The simplicity of treatment regimes is another factor determining a patient's decision, particularly if the diabetes management requires continuous treatment and monitoring. In today’s fast-paced lifestyle people want something simple and which does not interfere with their daily routines. Similarly for patients with T2DM, administering insulin was perceived by patients as troublesome as insulin requires cold storage, either in a refrigerator or a cooler bag or box. However, some patients were able to accommodate the difficulties. Feedback from patients showed mixed reactions. Two patients who had been on insulin therapy claimed they have adjusted well and were able to manage it even when on vacation. Another participant considered this as inconvenient and had this to say:

*“If I go travelling anywhere*, *I can’t take insulin along*. *If I go for courses*, *I don’t bring [it along] because there is no fridge at the venue*. *So I am forced to take oral medication*.*” (Female*, *52 years old*, *diagnosed with T2DM 3 years ago)*.

Keeping a balance between physical accessibility and affordability is another trade-off process. Accessibility to diabetes treatment in Malaysia is not the main barrier as there is flexibility for patients with T2DM to obtain their diabetes treatment either from public or private modern treatment settings or even from traditional and complementary medicine. When asked about access to quality healthcare, especially about affordability and availability, participants responded with mixed reactions. Despite the flexibility of physical access to treatment modalities, affordability of expensive treatment remained one of the important components in the patients’ decision making process.

Nonetheless, there was a patient who preferred to seek care from a private clinic because of a shorter waiting time to see doctors. Waiting time to see a doctor could be one of the predictors for physical accessibility to treatment. Patients may have easy access to public health facilities with only nominal fees, but the long waiting time pushes patients to seek treatment elsewhere. A family member of one patient explained the reason why his wife opted to seek treatment from a private clinic.

*“Public facilities are indeed cheaper*. *It only costs one Ringgit Malaysia [RM1] but we have to wait for almost half a day*. *We are not rich*, *but she [my wife] cannot stand for half a day*. *We have little money; we paid about RM40 [at a private clinic]*. *It was very fast [waiting time to see the doctor]*.*” (Patient’s husband*, *60 years old)*.

Balancing the cost and their perception on efficacy, side-effects, accessibility and simplicity of treatment regimens led patients with T2DM in this study to choose a particular treatment modality, while maintaining expectations and goals of treatment as the deciding factors.

This study also showed that patients who took traditional and complementary medicine were paying a significant cost for complementary treatment modalities to fulfil their ultimate desire to enhance their quality of life. The decision-making on the type of treatment modalities differed from patient to patient, depending on their personal evaluation and prioritization based on perception. There was no ‘best fit’ treatment modality for all patients or even for one patient. “Experimenting” of new treatment modalities took place continuously. The process of each episode of “experimentation” varied depending on the individual’s severity of symptoms and desired outcome.

### The Outcome of the “Experimentation”

In terms of their “experimentation” outcome, the responses varied, from positive to negative outcomes. The positive outcomes, as perceived by patients, included alleviation of symptoms and maintenance of quality of life that could sustain freedom from symptoms and better control of diabetes to avoid future complications. The negative outcomes, as stated by participants, were those that failed to produce any good result, or that did not result in any improvement in the illness. This negative result prompted patients to keep trying other treatment modalities until they achieved the desired outcome.

The study findings showed that patients with T2DM perceived the desired quality of life as the balance between functionality and adhering to the diabetes management plan, along with certain dietary freedom. Functionality included physical and social functioning, ability to perform work functions and carry out social obligations. Adhering to the diabetes management plan included compliance with prescribed management and treatment regimens. When the cycle of trial-and-error failed to achieve one’s desired outcome, the cycle was repeated with another modality of treatment, be it modern medicine, traditional treatment (e.g. seeking cure from a faith healer) or self-care, including home remedy. The phenomenon is well explained by the following excerpt shared by one participant:

*“We keep changing [different treatments] and trying [traditional herbs]; we want to see if the sugar level [glycemic control] will come down*. *Thank God [positive result]*.*” (Female*, *55 years old*, *diagnosed with T2DM 3 years ago)*.

## Discussion

This study aimed to develop a substantive theoretical model to explain help-seeking behaviour among patients with T2DM in a primary care setting. We have identified the core category “experimenting”, which has an explanatory grip on the help-seeking behaviour of patients with T2DM while manoeuvre through the healthcare system in Malaysia. Academically, “experimentation” has many descriptive definitions. The *Free Dictionary Online* defines it as ‘something to do with tests, to see if something works, or an attempt to try and improve it’. The search for positive outcomes was guided by an action of trial-and-error. “Trial-and-error” is defined as a method of reaching a correct solution or satisfactory result by appraising various means or methods (www.thefreedictionary.com, retrieved 1/3/2015). As the grounded theory approach[25], the core category “experimentation” is the concept that explains the help-seeking process; this model allows modifiability that works well and is relevant in different circumstances (adoption of different treatment modalities) or new contexts. For example, as diabetes progresses and complications develop, the patient’s life goals also change. The emerging illness symptoms and new unmet outcome because of changes in disease progression continue to trigger a series of “experimentations” to achieve their new goals. Thus, it is believed that the concept of “experimentations” can be extended to explain the help-seeking behaviour of patients with chronic illnesses beyond diabetes, which vary with the nature of the chronic illness, the disease progression and changes in health conditions over time.

The theoretical model (Fig 1) illustrates that the help-seeking process is not static, but involves dynamic and multifaceted processes that vary with each episode or each individual’s perception. The determinant factors for this model are more diverse than the earlier models described in the Health Belief Model, Healthcare Utilization Model, and Kroeger’s Model [26–28]. These models tend to describe help-seeking behaviour as a linear process, moving in one direction. This study supports the argument raised by Mackian S [29] that the earlier models do not have flexibility in understanding how people make decisions and the manoeuvres they undertake in the help-seeking process. While exploring the help-seeking behaviour among patients with T2DM, this study noted that each episode or experience of symptoms and complications is unique to individual patients. What was striking in this study is the emergence of the concept of “experimentation” while patients with T2DM manoeuvre through the healthcare system in their process of seeking diabetes treatment.

The model also demonstrated the whole process of treatment strategies and help-seeking, involving the “triggers” and information seeking to support the decision-making process. The model also explains that actions are only after careful evaluation of information obtained from various sources, including those from their social network that match with the person’s perception and experience. Diagnosis and expected patients personal outcome are the “triggers” in executing “experimentation” for treatment modality. There was a similarity of behaviour among people with urinary incontinence [30,31] and also patients with breast cancer [32].

Although the Health Belief Model noted that people balance their action against cost of treatment and its efficacy [33], the efficacy of treatment has always been perceived to be the overriding factor for the choice of treatment [34,35]. This study found that other characteristics may also affect patients’ decisions and choices. Availability of an excellent treatment may not guarantee patients’ accessibility to and affordability of the treatment and may even prevent them from getting the desired treatment. The patients in this study were able to prioritize or consider their choices of treatment modalities based on their life goals and perceived desired outcomes. For some, the high cost of treatment did not deter them from accessing the treatment when it was perceived to fulfil their goals.

### Strengths and Limitations

This study was planned to uncover the help-seeking process, and develop a substantive theory. The grounded theory methodology was chosen because it offers tools for an analytical depth beyond descriptive findings. The steps of this approach successfully rendered the analysis to construct “experimentation” and tested the concepts on subsequent interviews. Thus, the rigours of the analysis were demonstrated through confirming the analysis with subsequent data. The whole process was iterative as it utilizes constant comparison, theoretical sampling and theoretical saturation.

In addition, the richness and quality of data was obtained through the triangulation process of data collection using different methods of data collection and obtaining data from multiple sources, such as patients, family members as well as healthcare providers. The rigorous process of analysis and members checking process further ensured the quality of data and its interpretations [36].

However, the results of this study ought to be interpreted within the context of its limitations. The study was conducted in a primary healthcare setting with its inherent bias of participants selected for the study. The perceptions and help-seeking behaviour of the patients with T2DM in this study may not be generalised to the population of patients with T2DM in the country, who may or may not have differing help-seeking behaviour. Nevertheless, the core category of “experimentation”, can explain the help-seeking process in other contexts with or without modifications to other healthcare settings.

In addition, translation and interpretation of contents from multi-lingual interviews (as the interviews were conducted in Chinese, Malay and English languages) may not be context relevant. However, effort have been made to address this during the translation process as the researchers were fluent with all three languages used (English, Malay and Chinese) and they were familiar with the context of the study, thus ensuring the meaning remains the same. As with qualitative research, the emphasis was on an in-depth exploration of experience and perception. The findings of the study do not represent the average experience of patients with T2DM, but the theory developed and grounded in the data allows the generalization of the concepts and provides an explanatory power for help-seeking behaviour among patients with T2DM in a primary care setting. The substantive theoretical model has demonstrated in this study how it could fit [37] and be applied to the context of help-seeking behaviour among patients with T2DM and explain the same in other settings or other chronic illnesses.

## Conclusions

This exploratory study presents the substantive theoretical model of treatment strategies and how patients with T2DM maneuvering through the current healthcare system in selecting treatment for their illness. Essentially, patients experimented with different diabetes treatment options in order to achieve *their* goals, against which the outcomes of experimenting were compared. Patients continuously engaged in seeking information related to treatment characteristics to evaluate which treatment would produce the outcome they desired. Their effort of experimenting continued if the outcome did not match their goals. The mismatch triggered further experimentation. Thus, a responsive healthcare system needs to support patients’ “experimentation” process and provide a safe environment for their “experimentation”.
